# Endothelial Barrier Function and Leukocyte Transmigration in Atherosclerosis

**DOI:** 10.3390/biomedicines9040328

**Published:** 2021-03-24

**Authors:** Thijs J. Sluiter, Jaap D. van Buul, Stephan Huveneers, Paul H. A. Quax, Margreet R. de Vries

**Affiliations:** 1 Department of Vascular Surgery, Leiden University Medical Center, 2333 ZA Leiden, The Netherlands; t.j.sluiter@lumc.nl (T.J.S.); p.h.a.quax@lumc.nl (P.H.A.Q.); 2 Einthoven Laboratory for Experimental Vascular Medicine, Leiden University Medical Center, 2333 ZA Leiden, The Netherlands; 3 Sanquin Research and Landsteiner Laboratory, Leeuwenhoek Centre for Advanced Microscopy, Swammerdam Institute for Life Sciences, University of Amsterdam, 1066 CX Amsterdam, The Netherlands; j.vanbuul@sanquin.nl; 4 Department of Medical Biochemistry, Amsterdam Cardiovascular Sciences, Amsterdam University Medical Center, Location AMC, University of Amsterdam, 1105 AZ Amsterdam, The Netherlands; s.huveneers@amsterdamumc.nl

**Keywords:** endothelial barrier, endothelial cell junctions, endothelial dysfunction, inflammation, leukocyte transmigration, atherosclerosis, intraplaque-angiogenesis

## Abstract

The vascular endothelium is a highly specialized barrier that controls passage of fluids and migration of cells from the lumen into the vessel wall. Endothelial cells assist leukocytes to extravasate and despite the variety in the specific mechanisms utilized by different leukocytes to cross different vascular beds, there is a general principle of capture, rolling, slow rolling, arrest, crawling, and ultimately diapedesis via a paracellular or transcellular route. In atherosclerosis, the barrier function of the endothelium is impaired leading to uncontrolled leukocyte extravasation and vascular leakage. This is also observed in the neovessels that grow into the atherosclerotic plaque leading to intraplaque hemorrhage and plaque destabilization. This review focuses on the vascular endothelial barrier function and the interaction between endothelial cells and leukocytes during transmigration. We will discuss the role of endothelial dysfunction, transendothelial migration of leukocytes and plaque angiogenesis in atherosclerosis.

## 1. Introduction

The vascular endothelial lining is highly specialized and acts as a semi-confluent barrier controlling passage of fluids and migration of leukocytes from the lumen into the vessel wall. Under inflammatory conditions, endothelial cells (ECs) can assist leukocytes to leave the circulation to migrate into the underlying tissue to fight invading pathogens [1,2]. Under pathophysiologic conditions, such as chronic inflammation and atherosclerosis, ECs are activated and the barrier function of the endothelium becomes impaired, leading to uncontrolled leukocyte migration and vascular leakage [3,4]. Atherosclerosis starts with EC activation mainly due to lipid mediators such as oxidized low density lipoprotein (ox-LDL), which upregulates the expression of endothelial adhesion receptors such as intercellular adhesion molecule-1 (ICAM-1) and vascular adhesion molecule-1 (VCAM-1). Consequently, immune cells enter the subendothelial tissue initiating plaque formation. At later stages of plaque development, immune cells can also enter the plaque through vessels growing into the plaque [5,6]. These plaque neovessels are often immature with a limited barrier function [7] leading to increased immune cell transmigration and intraplaque hemorrhage. This results in plaque instability, ultimately leading to rupture and the progression of cardiovascular diseases, such as stroke, myocardial infarction (MI), or peripheral artery disease (PAD).

This review summarizes current knowledge on how the endothelium forms a semi-permeable barrier and how the endothelium assists the immune cells in their journey into the underlying tissue. We will discuss the role of endothelial dysfunction in plaque angiogenesis and its contribution to the development of atherosclerosis in different vascular beds.

## 2. The Endothelial Barrier Function

**Figure 1:**
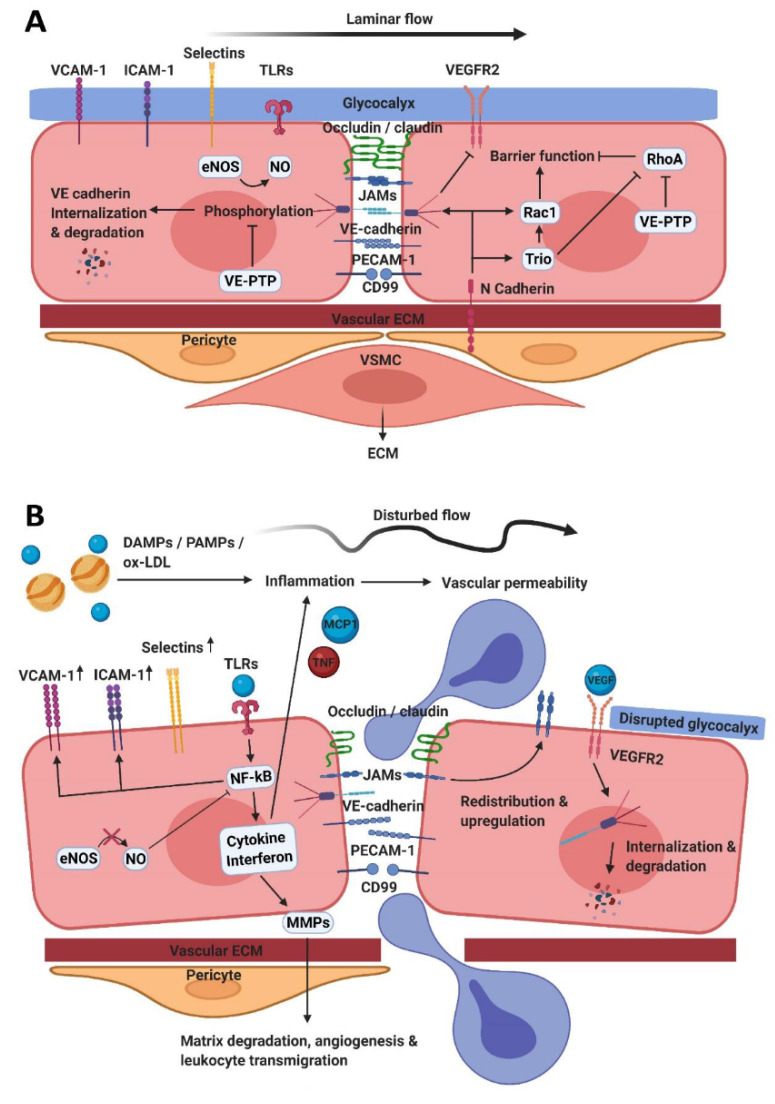
The endothelium under (patho)physiologic conditions. (**A**) Tight junctions, made by occludins, claudins, and JAMs, together with adherens junctions, formed by VE-cadherin, PECAM-1 and CD99 control endothelial barrier function by maintaining interendothelial junctions. Endothelial cells are connected to the vascular ECM, pericytes and VSMCs via N-cadherin and other interactions. N-cadherin activates Trio, Rac1 and also directly induces assembly of VE-cadherin junctions. Trio, as well as VE-PTP, inhibits RhoA. Additionally, VE-PTP prevents phosphorylation and subsequently degradation of VE-cadherin. NO is formed aiming to maintain a laminar flow together with the functional and intact glycocalyx. (**B**) Endothelial cells can become activated by disturbed flow and inflammatory mediators. VEGF activates VEGFR2 leading to internalization and degradation of VE-cadherin and TLR-activation results in NF-κB-activation. This mediates upregulation of adhesion molecules and cytokine/interferon production. This ultimately leads to leukocyte transmigration, vascular permeability, matrix degradation and angiogenesis. AJs—adherens junctions; DAMPs—damage associated molecular patterns; ECs—endothelial cells; ECM—extracellular matrix; eNOS—endothelial nitric oxide; ICAM-1—intracellular adhesion molecule-1; JAMs—junctional adhesion molecules; MMPs—matrix metallo proteinases; NO—nitric oxide; ox-LDL—oxidized low-density lipoproteins; PAMPs—pathogen-associated molecular patterns; PECAM-1—platelet endothelial cell adhesion molecule-1; TJs—tight junctions; TLR—Toll-like receptor; VCAM-1—vascular adhesion molecule-1; VE-cadherin—vascular endothelial-cadherin; VEGF—vascular endothelial growth factor; VEGFR2—VEGF receptor 2; VE-PTP—vascular endothelial protein tyrosine phosphatase; VSMCs—vascular smooth muscle cells.

The vascular endothelium lines the inner layer of the blood vessel and actively controls extravasation of fluids, ions, molecules, and leukocytes [8]. The integrity of the endothelium is maintained by intercellular junctions to prevent vascular leakage [9]. These cell–cell junctions consist of protein complexes that are part of the adherens junctions (AJs), gap junctions (GJs), tight junctions (TJs), and additional other adhesion receptors such as CD31/Platelet Endothelial Cell Adhesion Molecule—1 (PECAM-1), which can be disrupted during EC activation (Figure 1). GJs are formed by connexin-mediated transmembrane channels allowing direct communication between ECs via the passage of ions and small signaling molecules [10]. In contrast, AJs and TJs form adhesion structures that control paracellular permeability [8]. The organization of such EC junctions varies within the vascular tree depending on the tissue-specific function of the endothelium [11,12].

### 2.1. Tight Junctions

TJs control permeability of ions and small molecules and are most prominent in the blood–brain barrier (BBB) and the inner blood–retinal barrier where permeability is restricted [13]. TJs are mainly comprised of the receptors occludin, claudins and junctional adhesion molecules (JAMs) [14]. Claudin-5 is crucial for maintaining BBB-integrity in mice [15]. Adhesion molecules contribute to endothelial integrity through interaction with neighboring cells and induction of intracellular signaling, via their cytoplasmic domains and their extracellular domain [16]. TJ-proteins are linked to the actin cytoskeleton via the adaptor proteins Zonula Occludens (ZO) 1, 2, and 3 as well as other protein complexes [17]. Interestingly, bidirectional signaling has been found between ZO-1 and JAM-A. This regulates junctional localization of both molecules suggesting their involvement in upstream regulation of TJ assembly [18]. In addition, the assembly of TJs is dependent on formation of AJs [19] and changes in TJs are coordinated with changes in AJs [20].

### 2.2. Adherens Junctions

AJs are cadherin-based adhesions that provide mechanical strength to cell–cell junctions. Vascular Endothelial (VE)-cadherin has extracellular binding domains and a cytoplasmic domain that is connected to the actin cytoskeleton through interaction with catenins. VE-cadherin, together with PECAM-1-based adhesions, provides mechanical strength to the endothelial junctions [21,22]. Intracellularly, p120-catenin binds to the membrane-proximal cytoplasmic domain of VE-cadherin and promotes cadherin clustering by reducing turnover, whereas β/γ-catenins bind to the membrane-distal cytoplasmic domain, recruiting α-catenin which binds actin filaments and is therefore crucial for AJ stabilization [23,24]. Mice with a genetically engineered VE-cadherin-α-catenin fusion construct, unable to dynamically mediate this interaction, were resistant to vascular endothelial growth factor (VEGF)- and histamine-induced vascular leakage [25]. VE-cadherin promotes the formation of functional TJs through upregulation of claudin-5 [20], whereas the tight junction molecule JAM-C decreases endothelial integrity by targeting VE-cadherin, highlighting the complexity of the interplay between different junctional proteins [26]. The adhesion of VE-cadherin is controlled by phosphorylation and dephosphorylation on specific tyrosine residues [27]. In response to vascular permeability stimulators, such as VEGF and bradykinin, the tyrosine residues of VE-cadherin become phosphorylated (Y685 and Y658), resulting in instable AJs and an increased vascular permeability [28]. Interestingly, dephosphorylation of Y731 of VE-cadherin results in increased leukocyte transmigration [29,30]. It is important to note that leukocyte transendothelial migration (TEM) and vascular permeability are in fact uncoupled processes. During leukocyte TEM, vascular leakage is limited by a specialized endothelial-originated F-actin ring that acts as elastic strap surrounding the penetrating leukocyte [4].

One way of regulating the phosphorylation status of VE-cadherin is through the interaction with vascular endothelial protein tyrosine phosphatase (VE-PTP). VE-PTP binds through its extracellular domain to VE-cadherin and reduces tyrosine phosphorylation of VE-cadherin. This promotes the adhesive function of VE-cadherin by reducing its internalization independently of the phosphatase activity of VE-PTP. Thus, VE-PTP promotes the endothelial barrier and reduces vascular permeability [31,32]. VE-PTP can also influence Rho GTPase activity at AJs [32]. The family of Rho GTPases (e.g., RhoA) is crucial for the regulation of the endothelial barrier function as well as angiogenesis, as they control EC adhesion structures through cytoskeletal remodeling [33]. Rho GTPases are activated by guanine-nucleotide exchange factors (GEFs) and have different functions varying from improving cell-cell junction stability to mediating cell migration. VE-PTP inhibits binding of RhoGEF GEF-H1 to the small GTPase RhoA and decreases RhoA activity at AJs. As a consequence, local tension is lost resulting in more stable junctions and improved barrier function [32]. Trio, another RhoGEF, can bind to VE-cadherin during junction (re-)formation, and locally activate the small GTPase Rac1. Increased Rac1 activity at junction regions promotes the stabilization of VE-cadherin-based adhesions and thereby increasing the barrier function [34].

Another important endothelial cell-cell junction protein is PECAM-1. PECAM-1 is a transmembrane glycoprotein expressed on ECs, platelets, and several leukocyte-subsets [35]. Intracellular signaling occurs via phosphorylation of Ig-like immunoreceptor tyrosine-based inhibition motifs resulting in recruitment of Src Homology 2 phosphatases and interfering with tyrosine kinase pathways [36]. This affects various intracellular signaling molecules and pathways such as ICAM-1, interleukin (IL)-1β signaling, thereby affecting processes such as cell survival, shear stress and barrier integrity [37]. PECAM-1 can also promote endothelial junction stability through dephosphorylation of β-catenin [38]. It was found that ECs expressing PECAM-1 exhibited improved steady-state barrier function and more rapidly restored barrier integrity following perturbation, compared to PECAM-1 deficient ECs [35]. PECAM-1, together with VE-cadherin and VEGF receptor 2 (VEGFR2) forms a mechanosensory complex controlling responsiveness to flow [39]. The function of PECAM-1 in flow can impact downstream NF-κB activation, integrins, small GTPase RhoA signaling, actin polymerization, and thus the formation of stress fibers [39,40]. PECAM-1^−/−^ mice cannot activate NF-κB and downstream inflammatory genes in regions of disturbed flow [39].

### 2.3. Tissue-Specific Endothelium

Depending on the anatomical locations within the vascular tree, the endothelium can be relatively leaky (capillaries/venules) or not (arteries). In addition, depending on the organ-specific function of the endothelium, it can be continuous (e.g., brain) or fenestrated (e.g., kidney/glands), allowing for more or less extravasation respectively, depending on TJs and AJs [41,42]. Transit through fenestrated endothelium is limited to micro-molecules only and is controlled by e.g., blood flow, the basement membrane and glycocalyx [41]. The glycocalyx is a highly charged layer coving the luminal side of the endothelium functioning as a vascular barrier as well as a mechanotransducer regulating vascular tone [43]. The basal side of ECs is connected to the basement membrane, rich in laminin and type IV collagen, which strengthen the endothelium and can control extravasation of fluids in, e.g., the BBB [44]. In addition to paracellular permeability, ECs control transcellular transport of (macro)molecules such as lipoproteins.

Capillaries are also surrounded by pericytes which are multipotent perivascular cells. The EC-pericyte interaction is important to regulate EC proliferation, vessel tone and endothelial barrier function. Loss of pericyte coverage on the vascular endothelium is associated with excessive angiogenesis and BBB disruption, leading to increased plasma leakage [45,46]. Signaling between pericytes and ECs can occur through N-cadherin, which forms heterotypic adhesions between ECs and surrounding cells such as pericytes and VSMC and involves Trio signaling [47]. In addition, this intercellular communication also occurs via paracrine secretion of growth factors, such as VEGF, which increase leukocyte transmigration and angiogenesis [48].

The interstitial extracellular matrix (ECM), together with the basement membrane, forms the vascular ECM giving the endothelium structural and mechanical strength, necessary to resist hemodynamic forces on the vessel wall [49]. The endothelium also interacts with the vascular ECM. Collagens and fibronectin are the major components of the vascular ECM, in which also vascular smooth muscle cells (VSMCs) are embedded. ECM components can modulate the phenotype of vascular cells. During angiogenesis, ECs interact with type I collagen, resulting in upregulation of P-selectin and monocyte chemoattractant protein-1 (MCP-1) via ERK1/2 dependent mechanisms, which contribute to enhanced leukocyte attachment [50]. VSMCs contribute to the endothelial barrier function by providing resistance to mechanical stress, partly by synthesizing ECM, as well as regulating vascular tone [51]. In addition to the barrier function, the endothelium is responsible for regulating the vasomotor tone, maintaining a normal blood pressure, and consequently upholding laminar flow. Disturbed flow leads to increased permeability and is strongly correlated with atherosclerosis [52,53].

### 2.4. Vasomotor Function

Vasomotor tone is maintained by the release of numerous dilators and constrictors of which nitric oxide (NO) is the most important [54]. ECs metabolize L-arginine via the endothelial isoform of NO synthase (eNOS) to form NO, a process that is subject to both transcriptional and post-translational regulation [55]. Continuous exposure of the endothelium to risk factors such as hyperlipidemia, hypertension, smoking, shear stress, or inflammation leads to eNOS impairment [56]. Decreased NO availability results in inflammatory EC activation by upregulation of adhesion molecules such as ICAM-1 and VCAM-1, NF-κB mediated cytokine expression and disruption of the anti-thrombotic surface of the ECs. Other endothelium-derived vasodilators include prostacyclin and bradykinin. Bradykinin stimulates release of NO, prostacyclin, and the production of tissue plasminogen activator linking endothelial dysfunction to fibrinolysis. The endothelium also produces vasoconstrictor substances such as the very potent endothelin and angiotensin II which are counteracted by NO. Angiotensin II is also an antioxidant and enhances endothelin production and both stimulate VSMC proliferation, thereby contributing to atherosclerotic lesion formation [57].

## 3. Endothelial Activation and Leukocyte Transendothelial Migration

Ox-LDL, damage associated molecular patterns (DAMPS), and pathogen associated molecular patterns (PAMPs), as well as disturbed flow can activate the endothelium [58,59] in part by upregulation of Toll-like receptors (TLRs). TLR downstream signaling results in induction of the NF-κB-pathway, and downstream cytokine (a.o. TNF-α and MCP-1) and interferon production [60,61]. This, in turn, mediates the upregulation of adhesion molecules such as VCAM-1 and ICAM-1 [62,63,64]. Targeting this pathway could therefore decrease the inflammatory response and reduce leukocyte transmigration. NF-κB-pathway blockade resulted in decreased leukocyte TEM and TNF-α-induced expression of adhesion molecules [65,66].

Inflammatory cytokines and chemokines, such as TNF-α that are secreted by activated ECs can recruit leukocytes to sites of inflammation and initiate the process of leukocyte transmigration [67]. Furthermore, inflammatory activation of ECs triggers redistribution and upregulation of adhesion molecules such as JAM-A in vitro as well in vivo [68,69]. Upregulation and redistribution of adhesion molecules from EC junctions to the apical side of the EC mediates the inflammatory response [70].

Internalization of junctional adhesion molecules also regulates leukocyte TEM. In response to inflammatory stimuli, VE-cadherin-based junctions are disrupted, disassembled, and internalized from the membrane into the cytosol and ultimately degraded leading to increased vascular permeability and increased leukocyte transmigration [71]. In vitro it has been shown that ox-LDL promotes monocyte transmigration partially through downregulating VE-cadherin function and weakening of endothelial junctions [72].

Adhesion of leukocytes to and migration across the vessel wall into the underlying tissue occurs constitutively, but the frequency can increase in response to various stimuli and different conditions, such as endothelial activation, dysfunction, and inflammation [73]. Despite the variety in the specific mechanisms utilized by different leukocytes to cross different vascular beds, there is a general principle of capture, rolling, slow rolling, arrest, crawling, and ultimately diapedesis via a paracellular or transcellular route [1,74].

### 3.1. Finding Sites of Extravasation

Adhesion molecules expressed on ECs (e.g., selectins, CAMs), or chemokines and chemoattractants as well as shear stress can guide leukocytes to sites of extravasation. Presence of shear stress is mandatory for lymphocyte TEM in vitro [75]. Disturbance of flow results in disruption of VE-cadherin and β-catenin-mediated cell-cell junctions at cell borders [76]. Cytokines such as TNF-α released by circulating leukocytes stimulate neutrophil transmigration [77]. Prolonged exposure to inflammatory cytokines, upregulates expression of selectins, ICAM-1 and VCAM-1 on the EC surface [74]. For firm leukocyte attachment, activation of integrins is required. Lymphocyte function-associated antigen 1 (LFA-1, expressed by all leukocytes) and macrophage antigen 1 (Mac1, expressed by myeloid cells) are the most important integrins. Inflammatory cytokines, together with P- and E-selectin, can induce conformational changes of these integrins enabling leukocytes to interact with adhesion molecules promoting slow rolling and firm adhesion [78,79]. Lymphocytes, especially T cells, share some of the recruitment mechanisms with other leukocytes, but also have distinct mechanisms for extravasation. In response to specific major histocompatibility complex (MHC) molecules and cytokines, e.g., IL-12, T cells express different chemokine receptors and selectin ligands to acquire active and migratory phenotypes [74]. Interestingly, ECs can function as antigen presenting cells that also express costimulatory and co-inhibitory molecules as well as cytokines that can lead to activation of T cells [80].

Following firm adhesion, leukocytes crawl on the vascular endothelium, which has been suggested to enable adhering cells to find optimal sites for extravasation. Neutrophil crawling is strictly Mac1-dependent [81], whereas monocyte crawling can be Mac1- as well as LFA-1-dependent [82,83].

### 3.2. Opening Endothelial Cell Junctions

**Figure 2:**
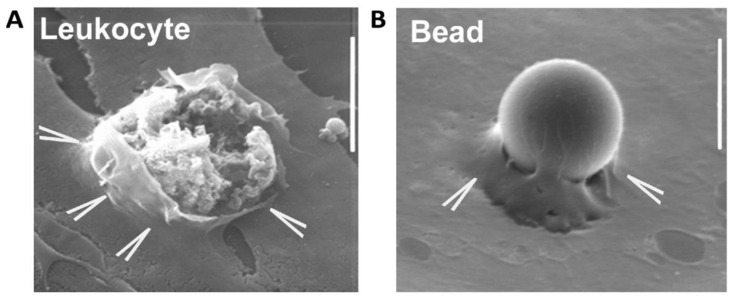
Leukocyte and static bead transmigration. Protrusive membranes (Endothelial protrusions, indicated by the arrowheads) arise from the apical endothelial surface and surround a leukocyte (**A**) or a static bead (**B**). The protrusions are believed to assist the leukocyte on its way through and limit vascular leakage. The image on the left may represent a transcellular event with the leukocyte on its way through the endothelial cell border, although the openings at the endothelial level may also suggest that this is a paracellular event. Scale bar: 10 μm.

Once leukocytes are guided to sites of extravasation, “docking structures” are formed that strongly correlate with leukocyte diapedesis and are therefore termed transmigratory cups [84,85]. These structures are enriched for ICAM-1 and VCAM-1 and consist of actin-rich membranes that can form microvilli-like protrusions, which “grasp” the leukocytes [86]. (Figure 2) The formation of these endothelial structures depends on intracellular ICAM-1 engagement and subsequent downstream activation of small GTPase RhoG through the RhoGEF SGEF [87]. Trio induces formation of docking structures through activation of RhoG and Rac1. Reducing Trio activity resulted in decreased leukocyte transmigration [88].

There are two ways for leukocyte TEM: either through the EC body, i.e., transcellular, or through intercellular junctions, i.e., paracellular. Although it has been observed in vitro and in vivo that most leukocytes migrate paracellularly, the transcellular route is also used [84,89]. Various cell surface receptors are involved in diapedesis including JAMs, VE-cadherin, and PECAM-1. Leukocytes expressing LFA-1 can bind to endothelial JAM-A and blockade of JAM-A reduced T cell TEM under both physiological and inflammatory conditions [90]. Moreover, JAM-A has been shown to be upregulated in human and mouse atherosclerotic plaques [91]. JAM-C interacts with leukocyte Mac-1 and prevents reverse transmigration of neutrophils [92] and, together with JAM-A, mediates polarization signals that facilitate neutrophil TEM [89,93].

As established, PECAM-1, is expressed on ECs, platelets, and leukocytes, but has distinct functions on each cell type. Antibodies blocking endothelial PECAM-1 increase vascular permeability [94] and it was therefore assumed that increasing PECAM-1 expression could reduce vascular permeability and thus leukocyte TEM by increasing cell–cell junction stability. However, PECAM-1 also inhibits activation of platelets and leukocytes [95]. PECAM-1-deficient mice have an excessive inflammatory response and therefore exhibit increased disease severity, [96,97] which is not only due to T cell hyperresponsiveness but also attributed to increased vascular permeability [98]. Increasing PECAM-1 expression on ECs, therefore, is an attractive target to inhibit leukocyte TEM, but the effects of PECAM-1 agonizing antibodies are most likely also derived from the immunomodulatory role of PECAM-1 on leukocytes rather than solely the effect on PECAM-1 expressed by ECs.

CD99 is a heavily glycosylated transmembrane protein that is expressed on leukocytes and ECs and is involved in leukocyte transmigration. Blocking of CD99 results in decreased migration of monocytes and neutrophils [99,100]. Specifically, blocking of CD99 resulted in arrest of both neutrophils and monocytes within EC junctions, whereas blockade of PECAM-1 resulted in arrest on the apical surface of the endothelium [99,100]. Different adhesion molecules act sequentially during diapedesis since deletion or blockade of ICAM-2, JAM-A, PECAM-1, and CD99 leads to arrest of leukocytes at different steps of TEM [99,101].

As previously established, VE-cadherin plays a central role in diapedesis. Preventing the dissociation of VE-PTP from VE-cadherin inhibits induction of vascular permeability and consequently leukocyte TEM [102]. Leukocyte-EC interaction can trigger this dissociation, thus promoting diapedesis. Interestingly, VEGF, can also trigger VE-PTP and VE-cadherin dissociation [103].

### 3.3. Leukocyte Migration into the Vessel Wall

After migration through the endothelial monolayer, which occurs within 2–5 min, leukocytes need to pass the endothelial basement membrane, which takes 20–30 min [104,105]. Although this indicates that passage across the basement membrane is a key rate-limiting step, this process is poorly understood [106]. It has been suggested that migration takes place at sites of the basement membrane that express low laminin and collagen IV [106,107,108]. Laminin 511 inhibits leukocyte transmigration by increasing junctional VE-cadherin and Laminin α5 was found to selectively inhibit T cell TEM through inhibition of T cell integrins [106,109]. Upon exposure to inflammatory cytokines and angiogenic factors, ECs produce matrix-degrading enzymes, such as matrix metalloproteinases (MMPs), resulting in decreased ECM protein content, which favors leukocyte TEM. For example, activity of a disintegrin and metalloproteinase (ADAM) 10 is required for leukocyte TEM [110]. Evidence also suggests that transmigrating leukocytes induce remodeling of the basement membrane via their proteases such as neutrophil elastase, but the exact mechanisms are not yet discovered [108,111].

### 3.4. Vascular Bed Specific TEM

Like leukocyte TEM under physiologic condition, TEM under inflammatory conditions is condition-specific, which is derived from EC heterogeneity. Single cell analysis of murine ECs from 11 tissues has identified 78 EC subclusters [112]. This analysis revealed that the tissue rather than the vascular bed determines EC heterogeneity, with capillary ECs exhibiting more tissue-specific variation than arterial and venous ECs. This is to be expected as exchange of nutrients primarily takes place at a capillary level. The recent discovery of EC heterogeneity is in line with prior findings that indicate tissue specific mechanisms for leukocyte TEM [113].

## 4. Atherosclerosis and Barrier Function

Endothelial dysfunction has proven to be an early marker for atherosclerosis [114]. In early stages of atherosclerosis, LDL molecules accumulate in the subendothelial region, where these molecules are then oxidized [115]. This is a major trigger for endothelial dysfunction, together with activation of TLRs by DAMPs. Targeting ox-LDL or modulation of TLRs clearly influences the influx of inflammatory cells in the vessel wall [60,116,117]. Ox-LDL, can cause upregulation and redistribution of, e.g., JAM-A which promotes lesion formation and leukocyte infiltration [68]. Genetic deletion of JAM-A on ECs reduced plaque size and monocyte and T cell presence in vivo, whereas genetic deletion of JAM-A on leukocytes did not decrease plaque size but did reduce monocyte and T cell presence. Moreover, transmigration of JAM-A^−/−^ monocytes over these JAM-A^−/−^ EC was decreased ex vivo compared to JAM-A^+/+^ monocytes. This highlights the cell-specific contributions of adhesion molecules.

In vivo EC activation and leukocyte transmigration can be inhibited by genetically or targeted inhibition of adhesion molecules. Inhibition of VCAM-1, either by genetic alteration or antagonizing antibodies, reduced plaque formation and infiltration of leukocytes [118,119]. Similarly, deficiency of ICAM-1 impaired lesion formation and soluble ICAM-1 correlated with disease severity [120]. Genetic deletion of SGEF resulted in decreased atherosclerosis by reducing docking structure formation (enriched with ICAM-1) and monocyte infiltration [121]. Genetic deletion of P- and E- selectin reduced plaque size and calcification in both early and advanced lesions [122]. Moreover, in the downstream processes, for instance, by EC specific inhibition of NF-κB reduced plaque formation, comparable effects such as decreased expression of VCAM-1 on ECs and cytokines in the aorta and impaired macrophage infiltration were observed [123].

It was found that the organization of VE-cadherin-based junctions at plaque endothelium was disorganized and frequently discontinued compared to normal endothelium [124]. Intriguingly, advanced plaque endothelium was found to be more organized than early atherosclerotic plaque endothelium and have improved luminal endothelial barrier function.

Polymorphisms of PECAM-1 are associated with endothelial dysfunction and adverse cardiovascular events in humans [125,126]. Interestingly, PECAM-1 has atherogenic or atheroprotective effects depending on the local hemodynamic environment. PECAM-1 deficiency protects from plaque formation in the inner curvature of the aortic arch (low flow) but enhances plaque formation in the descending aorta (high laminar flow) [127,128,129]. Inhibition of PECAM-1 decreased NF-κB-activation in response to atheroprone flow [128] and PECAM-1 deficiency reduced macrophage content, under low shear conditions [127].

Part of the extracellular domain of PECAM-1 expressed on T cells can be enzymatically shed upon T-cell receptor stimulation, whilst a juxta-membrane extracellular sequence remains expressed. This then can still be phosphorylated and in contrast inhibits T cell activation upon stimulation [130]. Loss of PECAM-1-expression on T-cells is associated with T cell hyperresponsiveness and atherothrombotic complications in humans and mice [131]. PECAM-1-agonizing antibodies decrease lesion formation, intraplaque hemorrhage, and intraplaque angiogenesis by reducing T cell activation and infiltration and increasing circulating regulatory T (T_reg_) cells [129,132]. These T_reg_ cells have anti-inflammatory and atheroprotective effects [133] and inhibition or depletion of T_reg_ cells is associated with atherosclerosis [134,135]. In contrast, other T cell subsets are known to enhance inflammation and exhibit atherogenic effects. T helper 1 (T_h_1) cells, for example, promote atherosclerosis by secreting pro-inflammatory cytokines such as IFNγ [136]. The use of immune checkpoint inhibitors, such as PD1-inhibitors, in cancer to activate T cells, has been shown to result in cardiovascular toxicity in humans [137]. In mice, PD1 deficiency resulted in increased atherosclerosis through exacerbated infiltration of T cells in the lesion [138].

In addition to the previously described adhesion molecules, platelets also play an important role in leukocyte TEM and early atherosclerosis formation. Platelets express various adhesion molecules, such as JAM-A and PECAM-1, and can interact with leukocytes [139]. Atherosclerotic plaque size was increased in platelet specific JAM-A^−/−^ mice with increased macrophage and T cell content. Moreover, these platelets exhibited increased binding capacity to leukocytes as well as increased inflammatory activity [140]. In addition to this distinct role in leukocyte recruitment, platelets are also needed to prevent bleeding during diapedesis. In mice with thrombocytopenia, neutrophil diapedesis was responsible for hemorrhaging demonstrating that not only leukocytes and ECs are involved in diapedesis but also other cells such as platelets [141].

### 4.1. Plaque Hypoxia

Once monocytes passage the endothelial barrier and reach the intimal space, colony-stimulating factor induces monocytes to phenotypically transform into macrophages and start taking up modified LDL particles [142]. Macrophages release reactive oxygen species (ROS) and inflammatory cytokines (a.o. TNF-α, MCP-1, IL-1, IL-6) which contribute to the continued recruitment and activation of other leukocytes. The activated macrophages also secrete MMPs which are clearly associated with plaque destabilization [143]. Macrophage activation involves a lot of metabolic processes resulting in a macrophage phenotype that is hypoxia inducible factor 1α (HIF-1α) dependent [144,145]. Upon transformation into foam cells, macrophages may undergo necrosis and thereby contribute to the necrotic core of advanced lesions. These advanced lesions are characterized by an increased vessel wall thickness resulting in regional limited oxygen exchange and thus hypoxic regions [146,147]. In a model of vein graft atherosclerosis, hypoxia replacement therapy resulted in enhanced vein graft patency and plaque stability via ROS mediated apoptosis of macrophages [148]. The oxygen-sensitive transcription factor HIF-1α is crucial in the adaptation to the local hypoxia status. HIF-1α induces transcription of hypoxia responsive genes such as VEGF, fibroblast growth factor, cytokines, and angiopoietins (Angs). Silencing of HIF-1α in macrophages reduces proinflammatory factors and increases macrophage apoptosis [149]. In contrast, deficiency of HIF-1α in antigen presenting cells induces T_h_1 polarization and aggravation of atherosclerosis via increased production of inflammatory cytokines [150].

### 4.2. Intraplaque Angiogenesis and Intraplaque Hemorrhage

**Figure 3:**
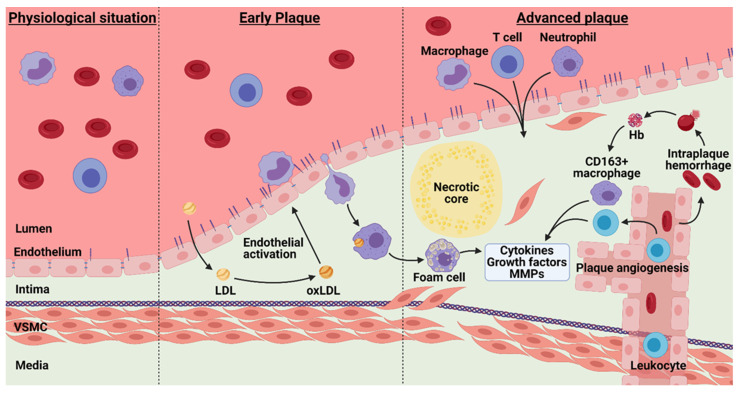
Pathophysiology of early and advanced atherosclerosis. Upon transcytosis and oxidation of LDL, the endothelium becomes activated leading to leukocyte infiltration into the vessel wall. This triggers thickening of the intimal layer, ultimately resulting in foam cell accumulation and necrotic core formation. Hypoxia induces intra-plaque angiogenesis, but these neovessels are often mature which leads to intraplaque hemorrhage and excessive extravasation of leukocytes. This results in production of cytokines, growth factors and MMPs, creating a continuous loop of plaque growth and ultimately plaque rupture. Hb—hemoglobin; LDL—low-density lipoproteins; MMPs—metallo matrix proteinases; oxLDL—oxidized low-density lipoproteins; VSMC—vascular smooth muscle cell.

**Figure 4:**
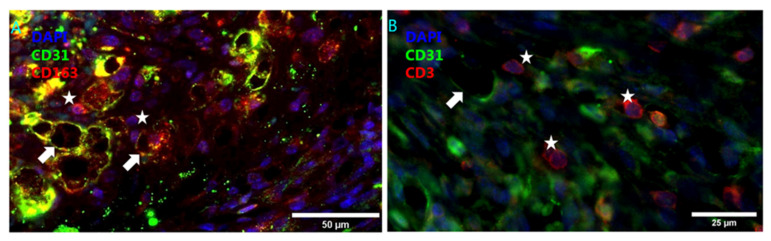
Inflammatory cell association with plaque neovessels. Inflammatory cells (indicated by stars) located near neovessels (indicated by arrows) in murine atherosclerotic vein grafts (**A**) Hemoglobin associated CD163+ macrophages localizing near neovessels, DAPI (blue), CD31 (green), and CD163 (red) (**B**) CD3+ T cells localizing near neovessels, DAPI (blue), CD31 (green) and CD3 (red).

Due to the hypoxia, plaque neovessels are formed through the HIF-1α-mediated transcription of angiogenic factors such as VEGF, to match the increased demand of oxygen in the plaque. VEGF, and activation of VEGFR2, promotes internalization of VE-cadherin and mediates the behavioral switch of ECs from a quiescent to an invasive phenotype leading to proliferation and migration of ECs into the plaque [151,152]. In turn, VE-cadherin limits VEGFR2 activation [153]. VEGF also induces ADAM10 and ADAM17 activity in ECs, mediating VE-cadherin cleavage [154]. It is thought that the ECs triggered by activation of VEGFR2 grow from the existing adventitial vasa vasorum into the atherosclerotic plaque [155]. In addition, Ang2 is upregulated and enhances angiogenesis by inducing detachment of pericytes to enable EC migration [156]. Moreover, Ang2 antagonizes Ang1, which promotes vessel maturation by recruiting pericytes, decreasing PECAM-1 and VE-cadherin phosphorylation. Ang2 also decreases basal and VEGF-induced permeability, thus protecting against plasma leakage and inhibiting leukocyte TEM [155,157]. Ang2, therefore, strongly compromises vessel maturation, yielding plaque neovessels with diminished structural integrity due to incomplete or absent endothelial junctions, basement membrane detachment and poor pericyte coverage rendering high susceptibility to vascular leakage [7]. Moreover, these ECs exhibit an activated and dysfunctional phenotype [7]. Indeed, intraplaque angiogenesis leads to intraplaque hemorrhage and plaque neovessels are co-localized with erythrocytes and immune cells. (Figure 3 and Figure 4A) [158]. In addition, erythrocytes are known to interact with the endothelium to facilitate and enhance influx of leukocytes into the plaque [159].

### 4.3. Angiogenesis Associated Macrophages

Once extravasated, erythrocytes lyse quickly, exposing hemoglobin which attracts monocytes and neutrophils into the plaque [160,161]. Macrophages can take up hemoglobin by CD163 leading to a distinct, alternative, non-foam cell macrophage phenotype [162]. These CD163^+^ macrophages are abundantly present in human and murine plaques expressing HIF-1α and VEGF, upregulating VCAM and associating with intraplaque angiogenesis and vascular permeability [163,164] (Figure 4B). Genetic analyses indicated that polymorphisms that result in increased CD163 expression and are a risk factor for plaque rupture [163]. Genetic deletion of CD163 in mice reduced plaque neovascularization, intraplaque hemorrhage and plaque progression. Regions in the plaque that exhibit active inflammation, determined by, e.g., macrophage content and MHC2-expression, show increased microvessel density in humans [165]. In advanced human lesions, ADAM17 expressing cells colocalize with CD68+-cells and ADAM17 is also associated with plaque-progression and neovascularization [153,166,167].

Extravasated inflammatory cells secrete growth factors, cytokines and MMPs, fueling intraplaque angiogenesis creating a continuous loop of plaque growth [168]. Intraplaque angiogenesis and consequently intraplaque hemorrhage are associated with plaque growth, instability, and ultimately rupture [169]. Targeting neovascularization may therefore stand as a promising approach to reduce atherosclerotic disease burden. Blockade of VEGFR2 in a murine vein graft atherosclerosis model decreased intraplaque hemorrhage, resulting in more stable atherosclerotic lesions with increased EC junctions with reduced macrophage content in vivo and increased pericyte coverage in vitro [164]. Adding to that, inhibition of basic fibroblast growth factor in the same vein graft atherosclerosis model resulted in decreased intraplaque angiogenesis and hemorrhage as well as reducing macrophage infiltration by reducing VCAM-1 expression [170]. Interestingly, myeloid ADAM17 deficiency is pro-atherosclerotic due to reduced shedding of TNF-receptor2 leading to sustained pro-inflammatory signaling. In contrast, endothelial ADAM17 deficiency is atheroprotective by mechanisms yet to be elucidated [171].

### 4.4. Statins

Statins are currently the principal drug in prevention of coronary artery disease (CAD) [172]. They hamper atherosclerosis progression by lowering LDL plasma levels, [173] but they have also been found to improve EC function and reduce inflammation [174,175,176]. Statins inhibit LFA-1 leading to decreased interactions with ICAM-1 and T cell activation [176] and reduce neutrophil transmigration by reducing Rho activity [117]. Intraplaque angiogenesis was found to be reduced in patients treated with atorvastatin compared to non-treated patients [177]. In mice, atorvastatin reduced neovessel formation whilst improving vessel maturation leading to decreased intraplaque hemorrhage in vivo. Atorvastatin was shown to increase VE-cadherin expression and pericyte coverage ex vivo and inhibit Ang2 release as well as VE-cadherin phosphorylation in vitro [28].

### 4.5. Atherosclerosis Heterogeneity

In line with EC heterogeneity, the degree of atherosclerosis differs in the various segments of the vascular tree. In CAD, for example, several studies have shown that proximal lesions are more frequently observed than distal lesions, possibly due to disturbed flow [178,179,180]. In addition, atherosclerosis also differs across different vascular beds. There is great distinction between intracranial (IC) and atherosclerosis in other vascular beds. Both can lead to severe ischemic events such as stroke, MI, and PAD.

IC atherosclerosis develops at a later age [181], but progression occurs more rapidly compared to the linear progression that is seen in atherosclerosis at other vascular beds [182]. Intracranial arteries have decreased permeability due to higher number TJs, which results in decreased susceptibility to inflammation [183]. In rabbits, ox-LDL impaired vaso-contraction and -dilatation in the carotid but not in the basilar artery, suggesting that IC arteries might be more resistant to ox-LDL [184]. In addition, IC arteries have a distinct morphology, which may also contribute to the initial resistance of IC arteries to atherosclerosis together with EC heterogeneity [112] may also contribute to the initial resistance of IC arteries to atherosclerosis. Vasa vasorum are initially absent IC arteries and develop only as a result of pathophysiological vessel wall thickening [185,186].

The variety between the arterial beds adds to the different forms of atherosclerosis (e.g., between coronary and peripheral atherosclerosis) [187]. In general, peripheral lesions have more calcifications but have a more stable phenotype compared to coronary lesions. In addition, peripheral lesions develop more slowly, but are more diffuse [188,189]. The increased susceptibility to atherosclerosis of the coronary arteries compared to peripheral arteries has been attributed to increased vasa vasorum density in coronary arteries [190].

## 5. Future Directions

Restoring endothelial barrier function and consequently reducing leukocyte transmigration could be an effective strategy to decrease atherogenesis. Moreover, reducing endothelial dysfunction would also improve quality of the neovessels present and consequently reduce intraplaque hemorrhage. Using local therapy targeting vascular bed specific abnormalities could prevent plaque progression with limited side-effects. Knowledge on endothelial dysfunction, plaque angiogenesis and vascular bed specific atherosclerosis is limited, and more research is needed to utilize its therapeutic potential to its fullest.

## Data Availability

Not applicable.
